# Embedded Optical Fibre with Fibre Bragg Grating Influence on Additive Manufactured Polymeric Structure Durability

**DOI:** 10.3390/ma15072653

**Published:** 2022-04-04

**Authors:** Magdalena Mieloszyk, Katarzyna Majewska, Artur Andrearczyk

**Affiliations:** Institute of Fluid Flow Machinery, Polish Academy of Sciences, Fiszera 14, 80-231 Gdansk, Poland; k.majewska@imp.gda.pl (K.M.); aandrearczyk@imp.gda.pl (A.A.)

**Keywords:** additive manufacturing, polymer, fibre Bragg grating sensor, tensile strength

## Abstract

Additive manufacturing (AM) polymers are applied in many branches of the industry due to the possibility of fast and accurate production of elements with various and complex shapes. Fibre Bragg grating sensors (FBG) are widely applied in structural health monitoring (SHM) systems. The main objective of this research is to perform analyses of the influence of embedded FBG sensors on AM polymer elements’ durability. Two polymers (M3 X and M3 Crystal) with different mechanical properties were analysed. The tests were performed on samples with FBG sensors embedded in (different alignment) and attached to the surfaces of the elements. Firstly, the samples were exposed to elevated or sub-zero temperatures under stable relative humidity levels. The strain in the samples was measured using fibre Bragg grating (FBG) sensors. The achieved results allow us to determine the relationships between strain and temperature for both materials and the differences in their mechanical response to the thermal loading. Then, the samples were subjected to a tensile test. A comparison of the tensile strength values was performed for the samples without and with embedded FBG sensors. The samples after the tensile tests were compared, showing differences in the mechanisms of failures related to the polymers and the thermal treatment influence on the material internal structure. Additionally, strain values measured by the FBG sensors were compared to the strain values achieved from the testing machine showing a good agreement (especially for M3 X) and indicating the differences in the materials’ mechanical properties. The achieved results allow us to conclude there is a lack of influence of embedded FBG sensors on the mechanical durability of AM polymers.

## 1. Introduction

Additive manufacturing (AM) techniques are recently very popular in many branches of industry due to the possibility of fast and accurate production of elements with various and complex shapes [1]. Among many materials, polymers have been recently widely applied [2] as they allow manufacturing structures both for everyday use and technical elements for testing prototypic solutions [3]. The variety of applications of AM elements causes them to be exposed to different mechanical loadings and environmental factors. The mechanical properties of AM samples are affected by both the unprinted material properties and the manufacturing process parameters. Among others, mechanical anisotropy being a result of the manufacturing process characteristic is the largest problem in AM elements [4].

There are a variety of optical materials that can serve as sensors for different physical or chemical parameters. One of the groups of materials is insulating materials that are commonly used for optical lenses or optical waveguides (optical fibres in telecommunication) [5]. Fibre Bragg gratings (FBG) can be inscribed in photosensitive fibres in almost any type of optical fibres, including silica core fibres and sapphire fibres [6]. The standard Bragg gratings written in silica core optical fibres are the most common and are applied as strain [7] or temperature [8] sensors. Another group of sensors are microstructured optical fibres. Among them are photonic crystal fibres consisting of cores and a periodic array of air holes as the cladding region [6]. Photonic crystals can be defined as structures in which the relative permittivity has a periodic variation in chosen orthogonal directions [9]. Photonic crystal sensors can be applied as refractive index sensing devices to distinguish for, e.g., pentane, ethanol, octane, ethylene glycol [10], nitrogen, or carbon dioxide [11]. FBG sensors can be also written on photonic crystal fibres for strain and temperature sensing, but the inscription process is time consuming [6].

FBG sensors are widely applied in structural health monitoring (SHM) systems [12] due to their advantages (e.g., small size and low weight, multiplexing capability, high corrosion resistance, and durability). The sensors’ properties allow them to be embedded into polymeric structures with limited influence on the material durability [13]. FBG sensors embedded into polymers are applied for designing special purpose sensors, e.g., high-sensitivity pressure sensors [14], biomedical pressure sensors [15], and simultaneous stress and temperature sensor [16].

The polymeric parts of the sensors (listed above) were manufactured using a conventional method—moulding [14,16]. The most important problems in applying conventional techniques to the manufacturing of precise elements are the dimensional stability (shrinkage) and occurrence of the residual strains due to phase change from liquid to solid in the entire volume of the mould. Such a problem can be overcome by applying an appropriate AM method.

AM techniques allow the manufacturing of functional components with embedded sensors that can be used in various monitoring techniques [17]. One of the most popular AM methods is fused deposition modelling (FDM) [18]. Bellacicca et al. [19] designed a prototypic AM electronic light sensor made out of thermoplastic with embedded electric circuits. Kanazawa et al. [20] manufactured a polymer cantilever beam with an embedded functional layer to be applied as a strain gauge. Hong et al. [18] used the FDM method to develop a pressure sensor based on the FBG sensor embedded into polylactic acid (PLA). Zhang et al. [21] designed a platform from PLA with FBG sensors to monitor plantar pressure distribution.

The fundamental mechanical test in mechanics and material science that allow determining the influence of embedded FBG sensors on AM polymers’ durability is the static tensile test. Wendt et al. [22] performed the tensile tests of PLA filaments and monolayer samples manufactured using the FDM method. Manufacturing defects were detected, e.g., differences in dimensions due to trajectory errors, irregular thickness, and gas bubbles. Despite, the manufacturing defects occurrence, the average variety of the maximum force was equal to 1.6%, while the modulus of elasticity was 4.2%. Plott et al. [23] analysed the tensile strength and failure modes in silicone samples manufactured using the FDM method. The influence of the manufacturing process parameters on the type and frequency of occurrence of the defects (e.g., elongated voids, infill tangency voids, and surface edges) and on the maximal ultimate tensile strength of the samples was observed. Hsueh et al. [24] analysed the influence of the FDM process parameters (the printing angle and the raster angle) and additional UV curing of PLA samples on their tensile strength. Additionally, UV curing increased the brittleness and decreased the elongation of the PLA material. Konigshofer et al. [25] analysed multimaterial jetting technology for a different combination of polymers used in medicine. The investigation was focused on the influence of mixing rates among the polymers on the tensile strength of the final material. Alaboodi and Sivasankaran [26] analysed mechanical properties of AM acrylonitrile butadiene styrene (ABS) samples with different levels of porosity (in a range of 0 to 30%). On the cubic samples, a compression test was performed for the purpose of determining mechanical parameters such as elastic modulus, plastic modulus, yield strength, and to determine their deformation mechanisms.

The aim of the paper is to determine the influence of embedded optical fibres (with FBG sensors) on AM polymeric materials’ durability. Polymeric elements are applied in engineering for prototypic solutions. In such cases, the possibility of embedding a sensor array into the element is very important, as the measurement results are then applied to optimise the designed construction prior to creating a final product that is typically manufactured from a more expensive material. Therefore, the presented investigation results are mostly focused on SHM system requirements and the influence of fibre optics with embedded FBG sensors on the manufactured element durability. Embedded FBG sensors being a part of SHM systems are applied for the measurement of strain values inside the structure under operational conditions. The analyses are performed on two materials with different mechanical properties manufactured using the same AM method—multi-jet printing (MJP). The durability of the materials was determined using the static tensile test performed on the samples after exposure to elevated or sub-zero temperatures.

The paper is organised as follows. Firstly, the manufacturing technique and samples with FBG sensors are described. Then, the experimental results related to thermal influence on the samples and the static tensile test are presented and discussed. Finally, some conclusions are drawn.

## 2. Manufacturing Technique

**Figure 1:**
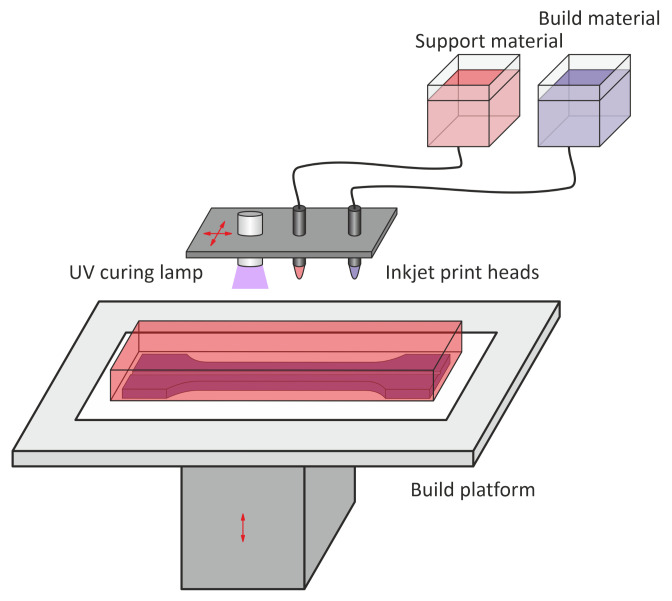
Multi-jet printing (MJP) printing principles.

The AM method applied for samples manufacturing is the MJP technique. It is one of the material jetting techniques used for manufacturing elements from UV-curable polymers [27,28]. It offers high accuracy of printed elements with complex shapes as the minimal thickness of the layer could be 16 μm [29]. This method was developed for the production of microturbine components at the Institute of Fluid Flow Machinery, Polish Academy of Sciences (IMP PAN) [30,31]. During the printing process, either photocurable plastic resin or casting wax material (supporting material) is deposited layer-by-layer. A scheme of the MJP printing principles is presented in Figure 1 with the moving directions of the print heads platform and the build platform lift marked. As is visible in the scheme, the material layers through printhead jets are distributed throughout the entire build platform. The next layer can be built after the previous is completely hardened. The printed object at the end of the process contains both the polymer element and the surrounding wax that is then removed using an oven or ultrasonic bath. Printing materials are available in different varieties, each defined by its specific physical and chemical characteristics. The inception of this technology arises from the need to provide high repeatability of details for printed elements [29]. The MJP technique application for manufacturing polymeric samples with embedded FBG sensors are described in detail in [29].

The samples were manufactured at the IMP PAN in a rapid-prototyping laboratory, using 3D printer ProJet HD 3500 Max (3D Systems, Rock Hill, SC, USA). The printing machine parameters are as follow. Its printing speed (XHD mode) is 4 mm/h and its resolution is equal to 750 DPI. The liquid polymer temperature in the printing head is equal to ca. 60 °C, while on the printing platform it is much lower and its value is ca. 40 °C. Therefore, there is no problem with residual strain occurrence due to heating. Additionally, the MJP technique [32] allows the manufacture of elements without voids, whose volume fraction affects the mechanical strength of the element as well as an embedded fibre optic sensor response to the environmental factors influence or mechanical loading.

## 3. Samples

Two materials (two categories of photopolymer materials—(meth)acrylate-based) were applied for manufacturing samples: M3 X and M3 Crystal. Both of them can be used for manufacturing an artificial scaffold for medical bone applications [26]. M3 X was also used for manufacturing elements for the Organic Rankine cycle (ORC) [30]. The selected material properties of M3 X and M3 Crystal are collected in Table 1 and Table 2, respectively. The data presented in the tables are from the manufacturer and the experimental tests performed at the IMP PAN.

**Figure 2:**
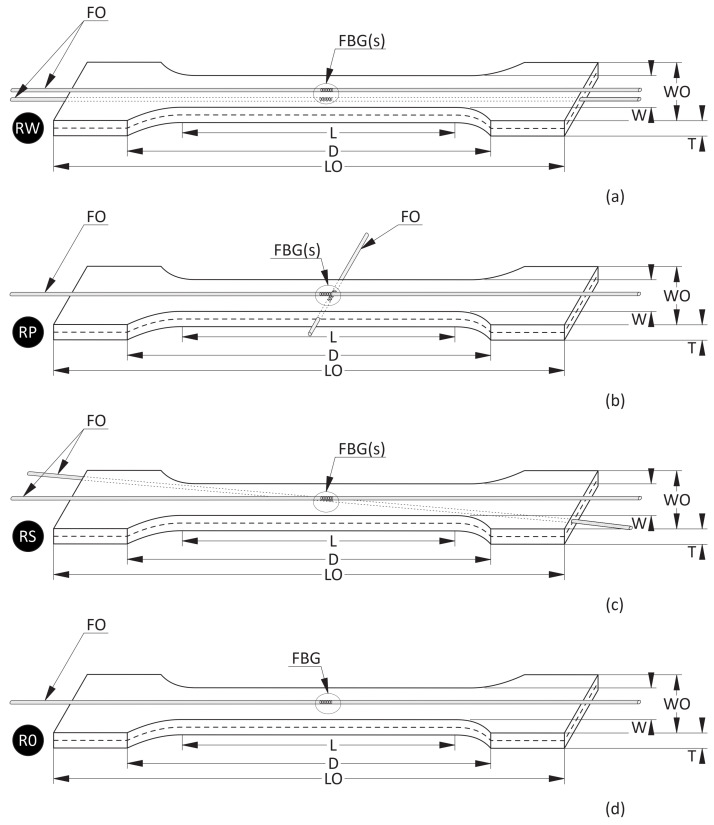
Schema of samples: (**a**) RW, (**b**) RP, (**c**) RS, and (**d**) R0. Symbols according to Table 4.

Four types of samples were manufactured. The samples schema are presented in Figure 2, while their dimensions are collected in Table 3. The samples shapes are related to tensile test requirements (Rule No. PN-EN ISO 527-1/1998). On one of the outer surfaces of each sample, an FBG sensor parallel to the main axis of the sample was attached. Such sensors were applied for strain measurements during the tensile test so they were located along the main loading axis. The applied adhesive was the same UV curable polymer as was used for manufacturing the particular sample. Additionally, fibre optics with FBG sensors were embedded in three of the sample types in the middle of the thickness of the samples. The samples’ symbols are related to the location of the embedded FBG sensor and are listed in Table 4. All embedded sensors are denoted by ‘*w*’ and the sensors attached to the surfaces by ‘*z*’. The sensors symbols are collected in Table 5. The used FBG sensors (Micron Optics, Atlanta, GA, USA) have a gauge length equal to 10 mm. The fibres were acrylate coated, whereas the coating around the measured grating lengths was removed. The used FBG sensors were the standard sensors written on silica core optical fibres as the investigation was focused on the future application of FBG sensors array in SHM systems installed in prototypic polymeric solutions.

## 4. Experimental Investigation

The experimental analyses were divided into two parts. The first was related to the determination of differences in the temperature influence on the samples materials, while the second was related to the tensile test.

### 4.1. FBG Sensors

The FBG sensors embedded in the samples and attached to the surfaces were applied for strain measurements. The total strain for all sensors was determined using the following equation:

(1) $$\varepsilon_{c}=\frac{\lambda_{m}-\lambda_{B}}{\left(1-\rho_{\varepsilon }\right)\lambda_{B}}$$

where indexes *m* and *B* are related to measured and base Bragg wavelengths, respectively. $\rho_{\varepsilon }$ is dimensionless effective photoelastic constant of the fibre core material [33].

The mechanical strain in the sample due to temperature and the relative humidity (RH) influence can be determined from the relationship

(2) $$\varepsilon_{m}=\varepsilon_{c}-\varepsilon_{f}$$

where $\varepsilon_{c}$ is the total strain measured by the FBG sensor, while $\varepsilon_{f}$ is a strain in the free FBG sensor due to temperature and RH influence on the sensor itself and the fibre optic material. The relationship between the FBG sensor wavelength and temperature under different humidity levels was determined experimentally. The experimental investigation and the process of the determination of characteristics of the FBG sensor in the form of the wavelength shift versus mechanical strain or temperature are presented in [29].

### 4.2. Thermal Tests

The thermal tests were performed in an environmental chamber MyDiscovery DM600C (Angelantoni Test Technologies Srl, Massa Martana, Italy) that allows for investigations in a range of temperatures from −75 °C to 180 °C with fluctuations of ±0.3 °C under different levels of RH ranging from 10% up to 98%, with ±3% fluctuations.

**Figure 3:**
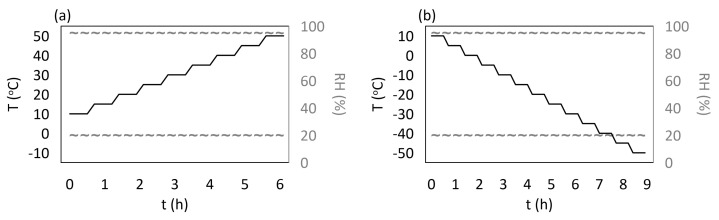
The thermal tests programs for temperatures: (**a**) elevated, (**b**) sub-zero.

The temperature influence test was divided into two parts: elevated and sub-zero temperatures. The tests programs are presented in Figure 3. The first measurements were performed at temperatures in a range from 10 °C to 50 °C and the second at temperatures in a range from −50 °C to 10 °C. In both cases, a 5 °C step between consecutive temperatures was assumed, and the measurements were performed for two stable values of the RH level: 20% and 95%. During the investigation, the samples were kept on a lattice shelf to allow them to expand in all directions. The temperature in the chamber was measured using an FBG temperature probe (Micron Optics, Atlanta, GA, USA). The FBG sensors measurements were performed using interrogator si425-500 (Micron Optics, Atlanta, GA, USA) with a measurement frequency equal to 1 Hz.

The total strain values were calculated using Equation (1). As the base condition, the temperature equal to 20 °C and the relative humidity equal to 20% were assumed.

**Figure 4:**
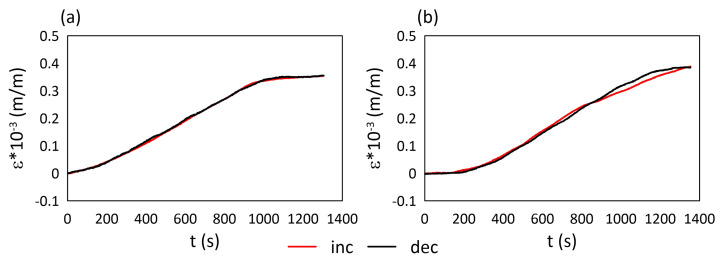
Strain hysteresis: (**a**) M3 X, (**b**) M3 Crystal; inc—temperature increasing, dec—temperature decreasing.

The heating process was performed in a way that allowed us to determine strain values for quasi-static conditions (steps). Both the heating and cooling processes were conducted at a very low speed (5 °C during 900 s). A comparison of the hysteresis of two analysed materials is presented in Figure 4. On both of them, parts related to temperature increasing and decreasing are highlighted. For M3 X, the strain hysteresis loop parts almost cover each other and the maximal strain difference is equal to 8 × $10^{-6}$ m/m. For M3 Crystal, the differences between the hysteresis loop parts are observable at the end of the heating process and the beginning of the cooling process. The maximal strain difference in this area is equal to 21 × $10^{-6}$ m/m.

**Figure 5:**
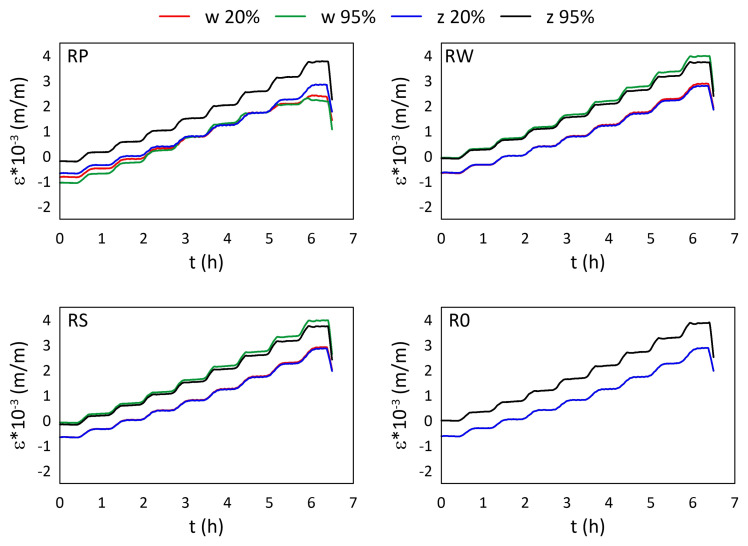
Strain measured by FBG sensors for M3 X samples under elevated temperatures; FBG sensors: *w*—embedded, and *z*—attached.

**Figure 6:**
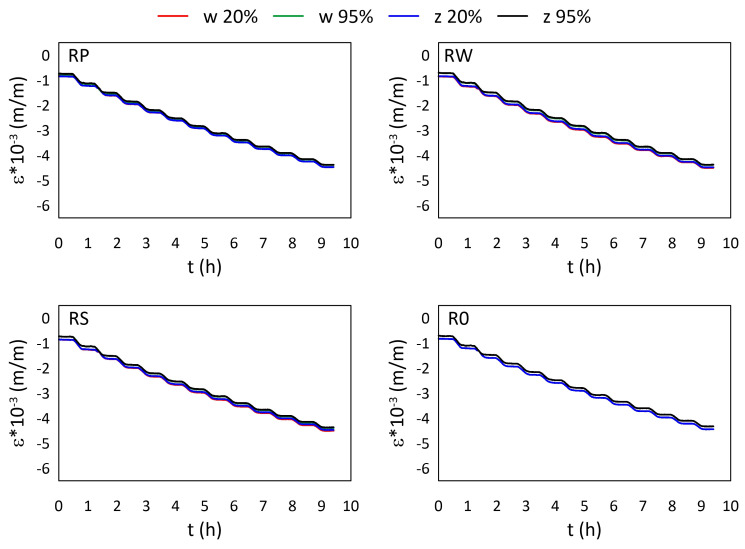
Strain measured by FBG sensors for M3 X samples under sub-zero temperatures; FBG sensors: *w*—embedded, and *z*—attached.

**Figure 7:**
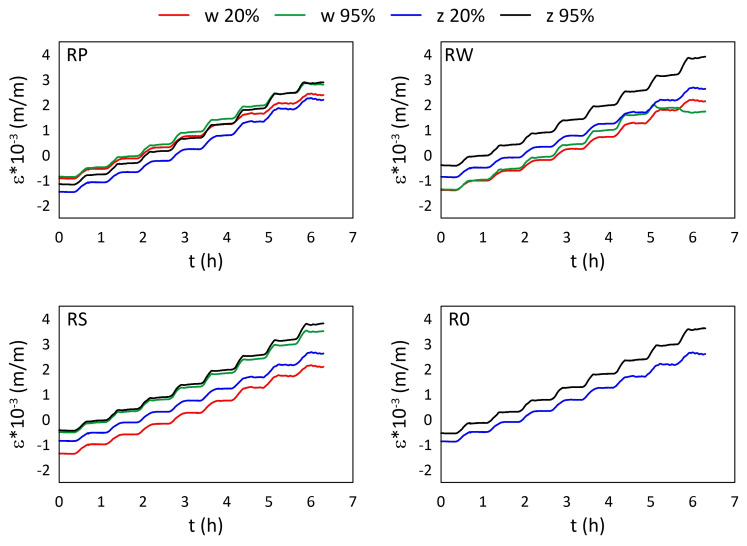
Strain measured by FBG sensors for M3 Crystal samples under elevated temperatures; FBG sensors: *w*—embedded, and *z*—attached.

**Figure 8:**
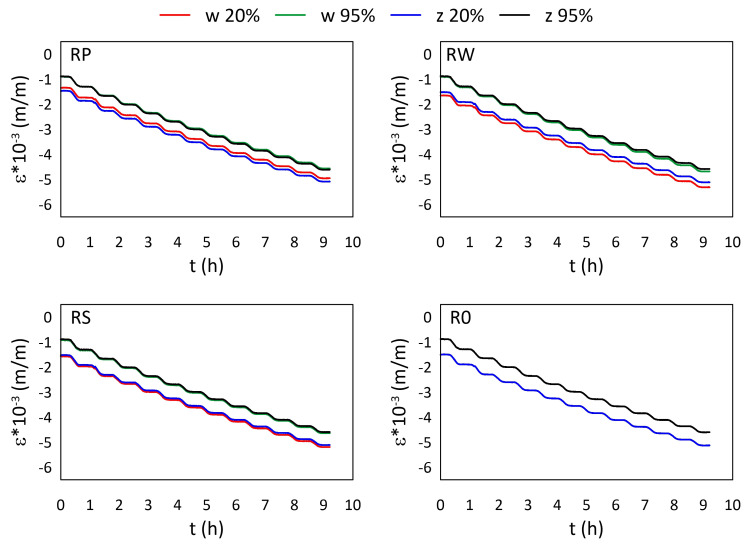
Strain measured by FBG sensors for M3 Crystal samples under sub-zero temperatures; FBG sensors: *w*—embedded, and *z*—attached.

The mechanical strain ε results for all M3 X samples from the thermal tests are presented in Figure 5 and Figure 6, while the mechanical strain ε results for all M3 Crystal samples from the thermal tests are presented in Figure 7 and Figure 8. The mechanical strain values were calculated using Equation (2). As it was presented in [29], the strain changes for free FBG sensors for the analysed range of temperatures are on $10^{-4}$ m/m level and almost linear. As the strain values level in the free FBG sensor due to temperature and humidity influences is one order of magnitude lower than the total strain in the samples, the removal of the environmental conditions influence on the sensors do not influence the strain curve shapes. Therefore, the mechanical strain curves have similar shapes as the total strain curves.

A comparison of mechanical strain ε values for all M3 X samples exposed to elevated temperatures are presented in Figure 5. For all sensors (except RPw) the influence of RH values is observable. The average strain differences between measurements performed for 20% RH and 95% RH are equal to $0.895\times 10^{-3}$ (m/m), while for RPw sensor the difference is one order of magnitude smaller. The strain values for 95% RH are higher, regardless of the temperature values. RWw sensor values for 40 °C and 50 °C are similar for both RH values. The RPw sensor behaves differently, probably due to its location inside the sample and the similarity between the gauge length (10 mm) and the sample thickness (12 mm).

A comparison of mechanical strain ε values for all M3 X samples exposed to sub-zero temperatures are presented in Figure 6. The achieved results are similar for all samples, regardless of the RH values and the location of the sensors. The average strain difference is equal to $0.110\times 10^{-3}$ (m/m).

A comparison of mechanical strain ε values for all M3 Crystal samples exposed to elevated temperatures are presented in Figure 7. Both FBG sensors (attached and embedded) from RP and RS samples show similar values for RH equal to 95%, regardless of the temperature. For RH equal to 20%, the strain values differ between embedded and attached sensors. For RW and RS, the attached strain values are higher, while for RP it is the opposite. Additionally, the strain values for RPw sensor determined for 20% RH up to 35 °C are similar (the average difference is ca. $0.173\times 10^{-3}$ (m/m)) to those measured for 95% RH (both RPw and RPz), while for higher temperatures the strain values are similar to RPz for 20% RH. In this case, the average difference is $0.510\times 10^{-3}$ (m/m). Strain values measured by RWw sensor for 95% RH are definitely smaller than those determined for the other embedded sensors under the same humidity level and are similar to the results achieved for 20% RH.

A comparison of mechanical strain ε values for all M3 Crystal samples exposed to sub-zero temperatures are presented in Figure 8. Contrary to M3 X material (see Figure 6), M3 Crystal is sensitive to humidity. Strain values determined for 95% RH are always higher than for 20% RH, regardless of the temperature, while the strain values for both sensors (embedded and attached) in the same sample are similar. The average difference between the strain values is equal to $0.562\times 10^{-3}$ (m/m).

For the purpose of determining the differences among the FBG sensors measurement results performed under the same thermal and RH conditions, the mean strain differences were calculated and collected in Table 6 and Table 7.

The mean strain differences between samples (for attached sensors ‘*z*’) were calculated using the following formulas

(3) $$\varepsilon_{mRW0z}=\frac{1}{m}\sum_{i=1}^{m}\left|\varepsilon_{RWz}-\varepsilon_{R0z}\right|$$

(4) $$\varepsilon_{mRWPz}=\frac{1}{m}\sum_{i=1}^{m}\left|\varepsilon_{RWz}-\varepsilon_{RPz}\right|$$

(5) $$\varepsilon_{mRWSz}=\frac{1}{m}\sum_{i=1}^{m}\left|\varepsilon_{RWz}-\varepsilon_{RSz}\right|$$

while for embedded sensors, (*w*) were calculated using the formulas

(6) $$\varepsilon_{mRWPw}=\frac{1}{m}\sum_{i=1}^{m}\left|\varepsilon_{RWw}-\varepsilon_{RPw}\right|$$

(7) $$\varepsilon_{mRWSw}=\frac{1}{m}\sum_{i=1}^{m}\left|\varepsilon_{RWw}-\varepsilon_{RSw}\right|$$

where *m* is equal to the number of intervals with stable temperature for the appropriate program (see Figure 3) calculated for sensors described in Table 5. For each case, the calculations were performed twice, separately for each RH value. Sensors from samples RW were chosen as the references, as both of them were laying along the main axis of the samples (compare schema Figure 2). The mean strain value for all samples (Table 6) was determined using the relationship

(8) $$\varepsilon_{mR}\left(20\%,\;95\%\;RH\right)=\frac{1}{n}\sum_{i=1}^{n}\left[\varepsilon_{mRWYz}\left(20\%,\;95\%\;RH\right)+\varepsilon_{mRWXw}\left(20\%,\;95\%\;RH\right)\right]$$

where *X* means *S* or *P*, while *Y* means *S*, *P*, or 0, and *n* is the number of measurements (number of sensors multiplied by the number of considered RH values). RPw sensor influence was calculated as a mean value of $\varepsilon_{mRWPw}$ separately for 20% and 95% RH. The mean strain values for all sensors except RPw were calculated using Equation (8) but without taking into consideration $\varepsilon_{mRWPw}$ separately for 20% and 95% RH.

A comparison of the mean strain differences among all samples is presented in Table 6. In both cases, RPw sensor is highlighted as it was embedded perpendicular to the main axis of the sample—see Figure 2. The differences in the behaviour of RPw sensor are well visible in Table 6. For both materials and for both thermal cases, the strain differences for RPw are the highest.

The strain differences between embedded (*w*) and attached (*z*) sensors were calculated using the following formula

(9) $$\varepsilon_{mRZwz}=\frac{1}{m}\sum_{i=1}^{m}\left|\varepsilon_{RZw}-\varepsilon_{RZz}\right|$$

where *Z* means *S*, *W*, or *P* (to achieve the proper sample symbol), and *m* is the number of measurements (number of intervals with stable temperature for the appropriate program—Figure 3). The mean strain differences between embedded (*w*) and attached (*z*) sensors for all samples (Table 7) were determined using the relationship

(10) $$\varepsilon_{mRwz}=\frac{1}{n}\sum_{i=1}^{n}\left[\varepsilon_{mRPwz}+\varepsilon_{mRWwz}+\varepsilon_{mRSwz}\right]$$

where *n* is the number of samples and $\varepsilon_{mRPwz}$, $\varepsilon_{mRWwz}$, and $\varepsilon_{mRSwz}$ are calculated for particular sample using Equation (9). The strain differences for RP sample were calculated using Equation (9) for *Z* equal *P*. The mean strain values for all samples except RP were calculated using Equation (10), taking into consideration *Z* means *W* and *S*. The calculations were performed for two RH values separately to analyse the possible water diffusion influence on the polymer materials and FBG sensors measurements.

As both sensors (attached and embedded) in the same sample are surrounded by the same polymers, the expected strain differences under the same conditions were relatively small. Based on the authors’ experience they should be on the $1\times 10^{-5}$ (m/m) level, while the measurement accuracy of FBG sensors is $2\times 10^{-6}$ (m/m). The mean strain differences between measurements performed by embedded and attached FBG sensors in one sample (RW, RP, and RS) were determined. The mean values calculated for those three sample types are collected in Table 7. It is well visible that, for M3 X exposed to elevated temperatures at 20% RH and sub-zero temperatures at 20% RH and 95% RH, the achieved results are on the expected level. Such differences for elevated temperatures and 95% RH are higher. They were probably related to the dissimilarity influence of humidity on the polymeric material: stronger on the element surface and weaker in the middle. For M3 Crystal, higher differences between embedded and attached sensors are observable than for M3 X. This is especially visible for elevated temperatures. There are two main probable reasons. The first is related to the structural changes in the material due to the maximal elevated temperatures in the test. The remaining effect is visible for all sensors for temperatures higher than 40 °C in Figure 7. Such temperatures are ca. 5 °C smaller than the heat distortion temperature of the material (Table 2). The other reason can be the relative humidity levels and the diffusion processes of water into the polymer. The humidity influence is smaller for sensors (RWw, RSw) embedded in the middle of the samples than on the sensors (RWz and RSz) attached to the samples’ surfaces. The same process caused the strain differences between RPw and RPz to be smaller. RPw sensor location and its direction according to the main axis of the sample (Figure 2) increase the possibility of water diffusion along the embedded fibre optic due to the capillary effect occurrence. The process was described in more detail in [34] for FBG sensors embedded in a glass fibre reinforced polymer sample.

Then the relative strain differences for embedded (*w*) and attached (*z*) sensors were calculated using the relationships

(11) $$E_{w}=\left|\frac{\varepsilon_{RXw}-\varepsilon_{RWw}}{\varepsilon_{RWw}}\right|$$

(12) $$E_{z}=\left|\frac{\varepsilon_{RYw}-\varepsilon_{RWw}}{\varepsilon_{RWw}}\right|$$

where *X* means *S* or *P* while *Y* means *S*, *P*, or 0. Sample RW was chosen as the reference value as the embedded sensor (RWw) was located along the main axis of the sample.

The mean values were calculated for each sensor and the results, in the form of percentage differences, are collected in Table 8 and Table 9 for embedded and attached sensors, respectively.

For M3 X material, the percentage difference was ca. 2% except RPw sensor under 95% RH. In such a case, the most possible reason is the influence of the water diffusion into the material and the capillary effect of the sensor that length is similar to the sample width. For M3 Crystal material, the average strain differences are very high for the elevated temperatures in comparison to the sub-zero temperatures. The observable effect is probably a result of lower heat distortion temperature (Table 2) and its influence on the mechanical properties of the material—disturbance of the proper material response to the temperature loading.

In the next step, the relationship between strain and temperature was determined using the following formula:

(13) $$\varepsilon_{Tj}=\frac{1}{n}\sum_{i=1}^{n}\varepsilon_{n}\left(T_{j}\right)\;for\;j=1,\cdots ,m;$$

where temperature level $T_{j}$ is related to averaged temperature value from *n* points for stable temperature conditions lasting 300 s and *m* equal to the number of intervals with stable temperature for appropriate program (see Figure 3). The averaged calculation error for temperature was 0.35 °C, whereas that for the strain was 8 × 10^−6^ m/m. The measurement accuracy of the interrogator is equal to 1.0 × 10^−6^ m/m, so the differences are neglected. The calculation errors were determined considering all measurements performed during the investigation for both materials jointly.

The resulting relationships are the thermal characteristics of materials that are useful for mechanical engineers and applied during the determination of strain in structures due to mechanical loading of structures operating under different environmental conditions. An example is the determination of strain originated from sea loading acting on a hull of a fast patrol boat for which the thermal characteristics of the material were defined based on a composite panel with the same structure as the hull [35].

**Figure 9:**
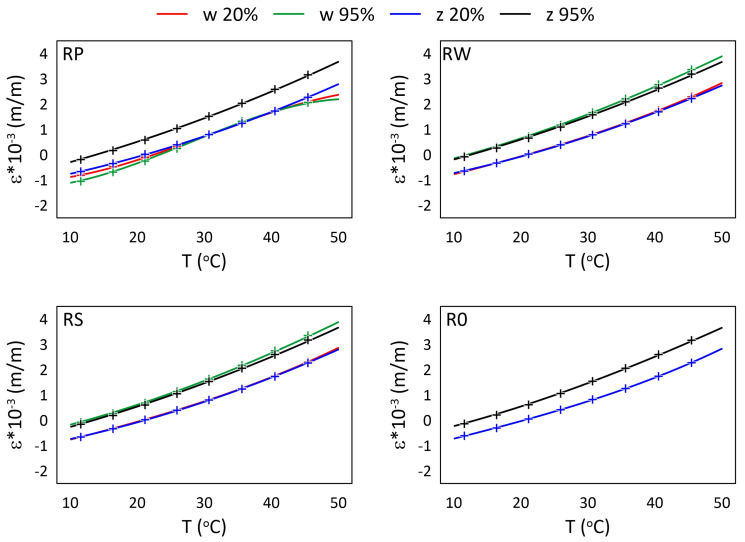
Strain for M3 X samples under elevated temperatures and selected RH (20%, 95%) levels; crosses—measured values, continuous line—approximation.

**Figure 10:**
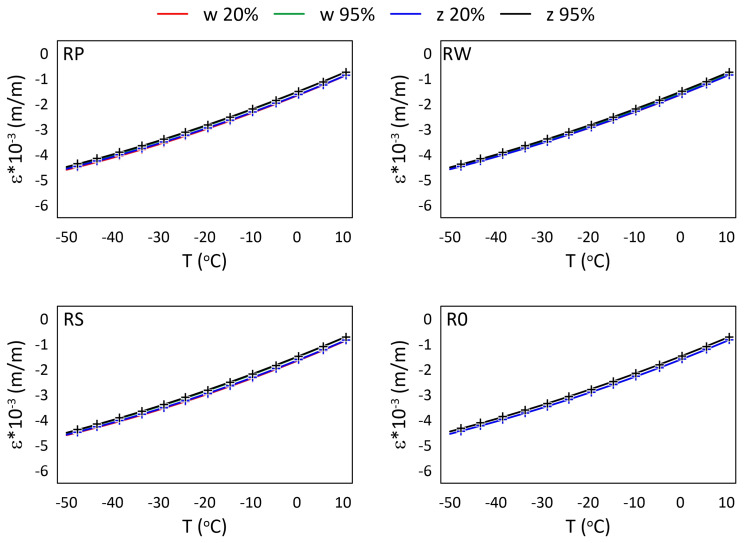
Strain for M3 X samples under sub-zero temperatures and selected RH (20%, 95%) levels; crosses—measured values, continuous line—approximation.

**Figure 11:**
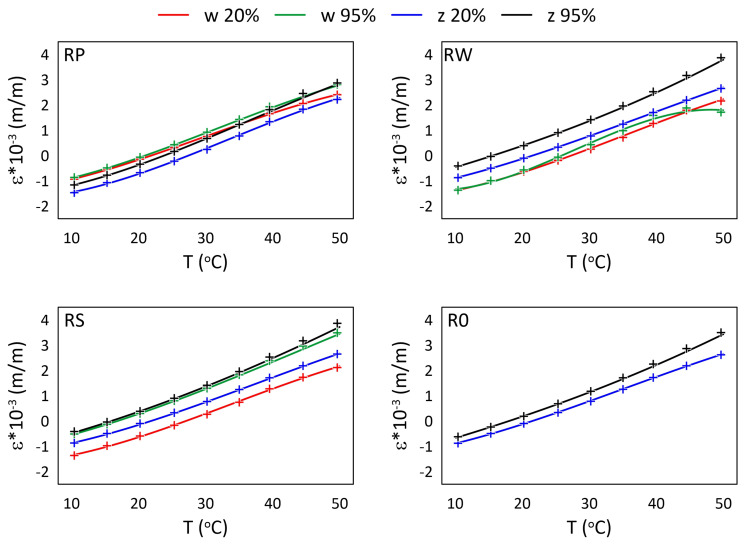
Strain for M3 Crystal samples under elevated temperatures and selected RH (20%, 95%) levels; crosses—measured values, continuous line—approximation.

**Figure 12:**
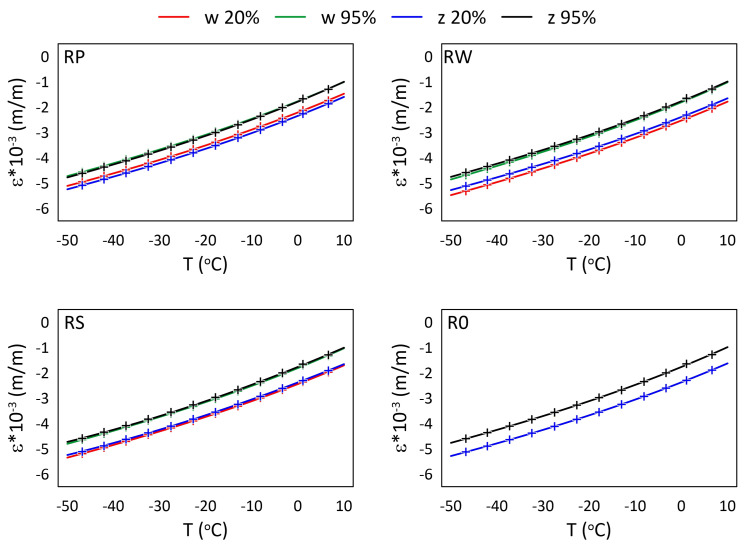
Strain for M3 Crystal samples under sub-zero temperatures and selected RH (20%, 95%) levels; crosses—measured values, continuous line—approximation.

The relationships between strain values and temperature for all M3 X samples are presented in Figure 9 and Figure 10, while for all M3 Crystal samples in Figure 11 and Figure 12. The approximated curves for all sensors except RP${}_{w}$ have similar shapes and can be written in a form of a polynomial; analogically, as was presented in [29].

For M3 X material under elevated temperatures (Figure 9), it is well visible that RPw sensor is more sensitive on RH than the other sensors. It is probably the effect of the sensor direction according to the main axis of the sample (Figure 2). Generally, the strain values are similar for the same humidity levels regardless of the locations of the sensors (embedded or attached). It well agreed with the assumption related to using the same polymer for manufacturing samples and attaching sensors on their surfaces.

For M3 X material under sub-zero temperatures (Figure 10) the determined relationships are similar for all sensors from all samples, regardless of the RH values. The water diffusion coefficient for the polymer is likely quite low. Therefore, the time when the sample during the thermal test is exposed to above zero temperatures (see Figure 3b) is too short to allow enough water to diffuse into the material to change its thermal response in the FBG sensors location.

A comparison of strain values measured by embedded FBG sensor (corresponding to RWw sensor in the paper) and calculated numerically (finite element method) was presented and discussed in [36]. For above zero temperatures, the average percentage error was ca. 3%, while for sub-zero temperatures it was ca. 5%.

For M3 Crystal exposed to elevated temperatures (Figure 11), the differences among sensor measurements significantly increase with the temperature. When the temperature is close to the heat distortion temperature (see Table 2) the differences between strain values determined for the sensors are the highest. It is especially visible for sensors RWw and RPw, where the strain distortion is also observable as the curves shape changes. Furthermore, the RH effect is visible as the increase in the distances between the curves (determined for the same sensor under 20% RH and 95% RH for temperatures higher than 40 °C).

For M3 Crystal exposed to sub-zero temperatures (Figure 12) the influence of RH is visible. For all sensors, the strain values for 95% RH are higher than for 20% RH. The water diffuse coefficient for M3 Crystal is likely higher than for M3 X. Therefore, the time when the samples are in the above zero temperatures (Figure 3b) is long enough to allow the polymer to absorb enough water to make its influence noticeable on the thermal strain measurements.

**Figure 13:**
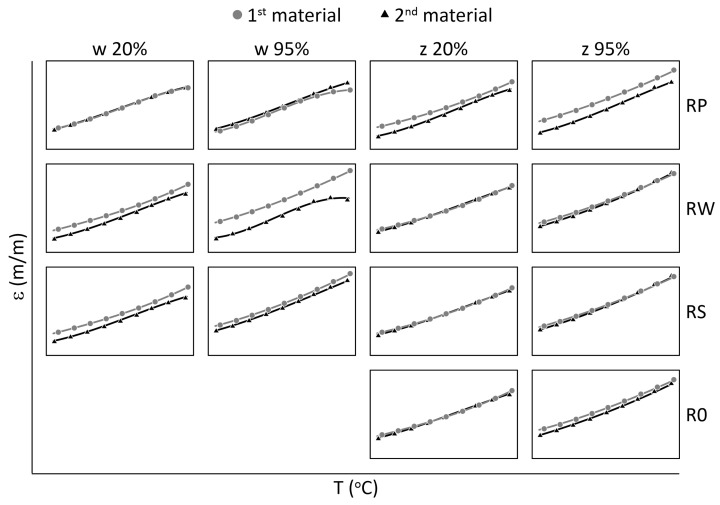
A comparison of strain trends for M3 X (First Material) and M3 Crystal (Second Material) samples under elevated temperatures.

**Figure 14:**
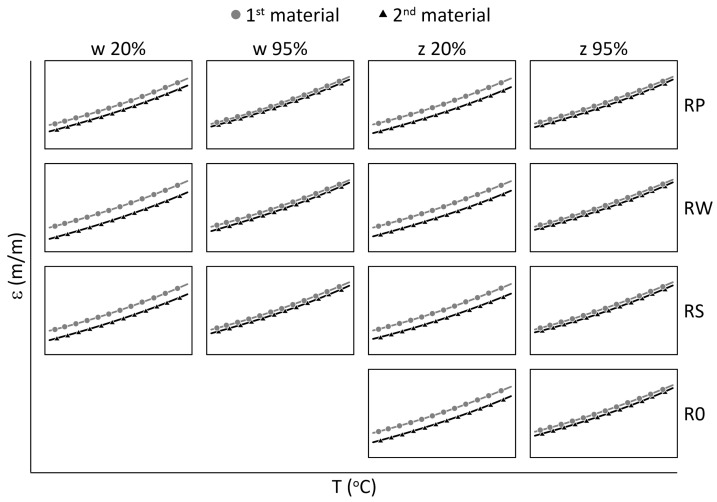
A comparison of strain trends for M3 X (First Material) and M3 Crystal (Second Material) samples under sub-zero temperatures.

For the purpose of better understanding the differences between materials, a comparison of their behaviour under different temperatures and RH levels are presented in Figure 13 and Figure 14 for elevated and sub-zero temperatures, respectively.

The curves presented in Figure 13 for elevated temperatures show that the relationships between materials are strongly related to the FBG sensors’ locations. As described earlier, the influence is higher for M3 Crystal material. Despite this, for the majority of cases, the strain values for M3 X are higher than the corresponding M3 Crystal strain values. For the purpose of better visibility of the materials’ strain relationships, the mean values of strain differences were calculated and collected in Table 10. For each case, one sensor behaves differently, and the strain differences determined for it was significantly lower or higher than for the others. Such sensors with their maximal or minimal strain values are listed in the table. For almost all cases the sensors are from the RP sample type. Due to the definitely different behaviour of the sensors, they were not used for determining the mean strain differences between materials.

The strain differences between the strain curves determined for two considered materials (M3 X and M3 Crystal) were calculated using the following formula:

(14) $$\varepsilon_{mMRZx}=\frac{1}{m}\sum_{i=1}^{m}\left|\varepsilon_{M1RZx}-\varepsilon_{M2RZx}\right|$$

where *M* is related to material (*M*1 means M3 X and *M*2 means M3 Crystal), *Z* means *S*, *W*, *P*, or 0 and is related to the samples, *x* is related to the locations of the sensors (‘*w*’ embedded or ‘*z*’ attached), while m is the number of measurements (number of parts in programs (Figure 3) with stable temperature values). The calculations were performed for each RH level separately. The mean strain differences for each RH level and the locations of the sensors (embedded and attached) were calculated using the following formula

(15) $$\varepsilon_{mMRx}=\frac{1}{n}\sum_{i=1}^{n}\varepsilon_{mMRZx}$$

where *n* is the number of samples and *Z* is related to all samples used in determining the average strain value. The calculations are performed according to columns of graphs presented in Figure 13 and Figure 14 for elevated and sub-zero temperatures, respectively. For elevated temperatures, the mean values were calculated for all sensors except the one highlighted in the bottom row in Table 10. The $\varepsilon_{mMRZx}$ value determined for the sensor using Equation (14) is listed in the table in the row with the maximal or minimal values. Except for one case, the sensors are from the RP sample type.

The curves presented in Figure 14 for sub-zero temperatures show a strong relationship between strain and RH values. For all cases, the strain values for M3 X are higher than corresponding to them M3 Crystal values. The mean values collected in Table 11 highlight the RH influence on M3 Crystal. Regardless of the sensor location, the mean differences between strain values in materials are equal to ca. 0.64 × 10${}^{-3}$(m/m) and 0.18 × 10${}^{-3}$(m/m) for 20% RH and 95% RH, respectively.

### 4.3. Tensile Test

**Figure 15:**
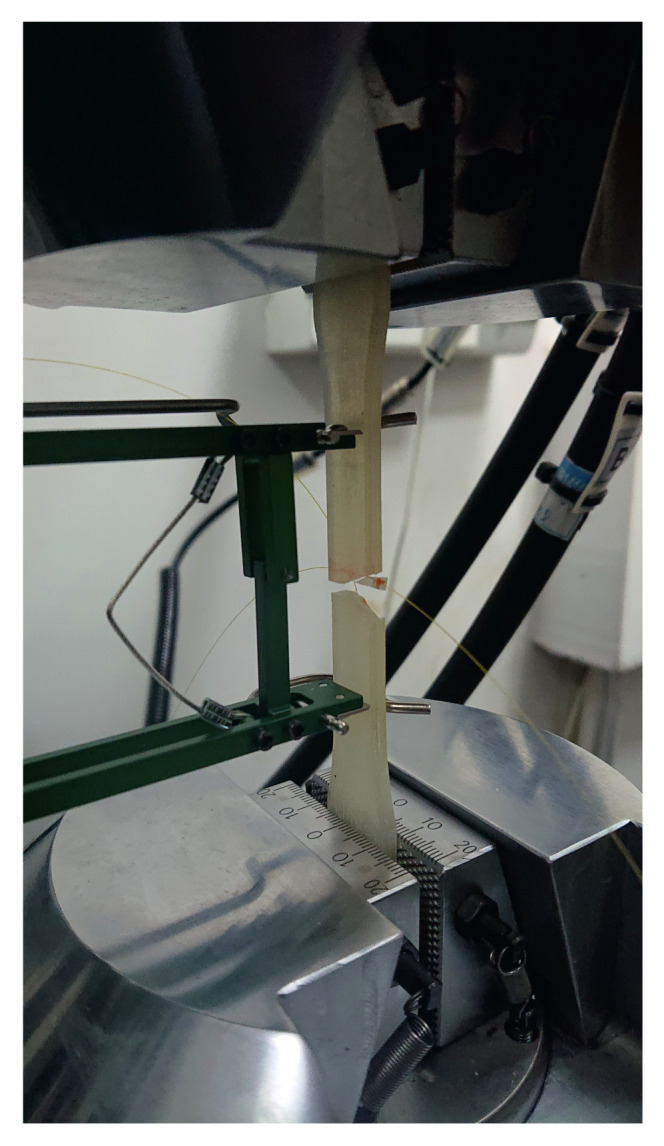
The sample mounted into the tensile machine jaws.

All samples described above were then mechanically tested by the tensile test. Its aim was to determine the embedded fibre optic influence on the durability of the polymeric materials. The tensile tests were performed on universal static-dynamic testing machine HT-9711-25 (Hung Ta, Gwangmyoung Techno Park, Korea). A photograph of the sample mounted into the tensile machine jaws is presented in Figure 15.

**Figure 16:**
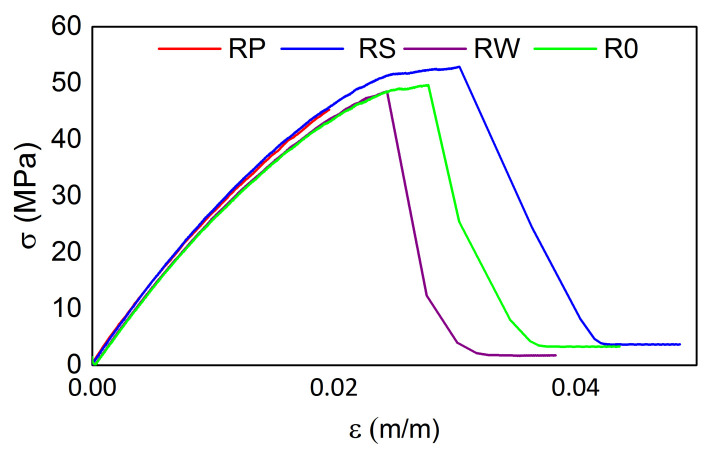
Tensile test results for M3 X samples after exposition on elevated temperatures.

**Figure 17:**
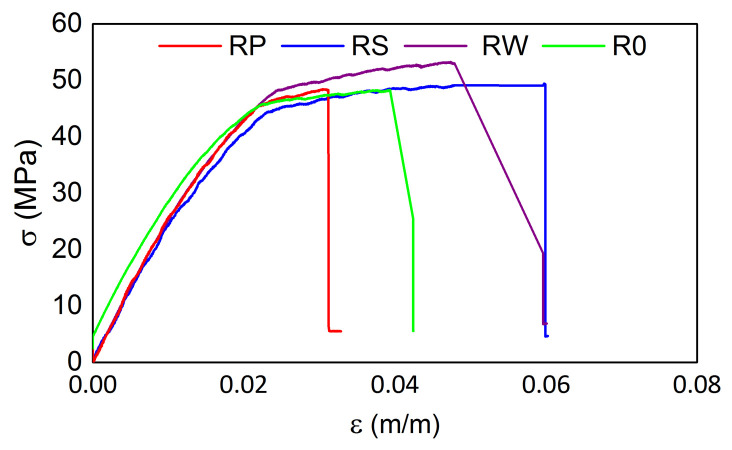
Tensile test results for M3 X samples after exposition on sub-zero temperatures.

The tensile test results from the testing machine for all M3 X samples are presented in Figure 16 and Figure 17 for the samples after exposition to elevated and sub-zero temperatures, respectively. The tensile strength values are similar for all samples regardless of the previous thermal treatment parameters while the strain values differ among samples. The mean tensile strength value is equal to ca. 49 MPa, while the mean strain value is equal to 0.026 m/m. For both cases, samples RP show the smallest strain values (0.020 m/m). It is likely caused by the embedded sensor location (Figure 2).

**Figure 18:**
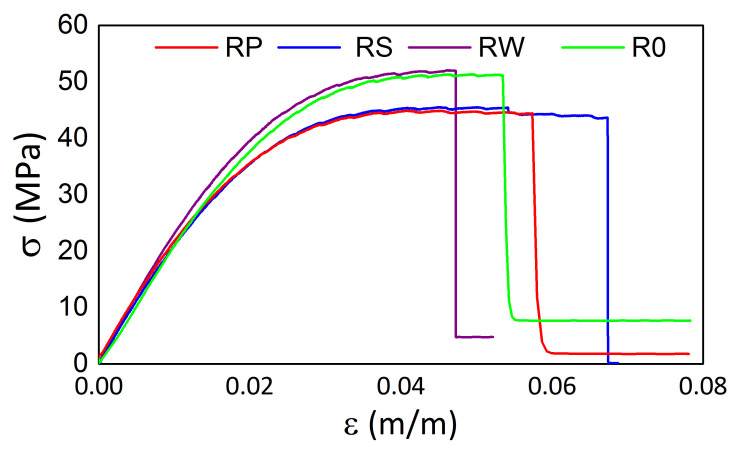
Tensile test results for M3 Crystal samples after exposition on elevated temperatures.

**Figure 19:**
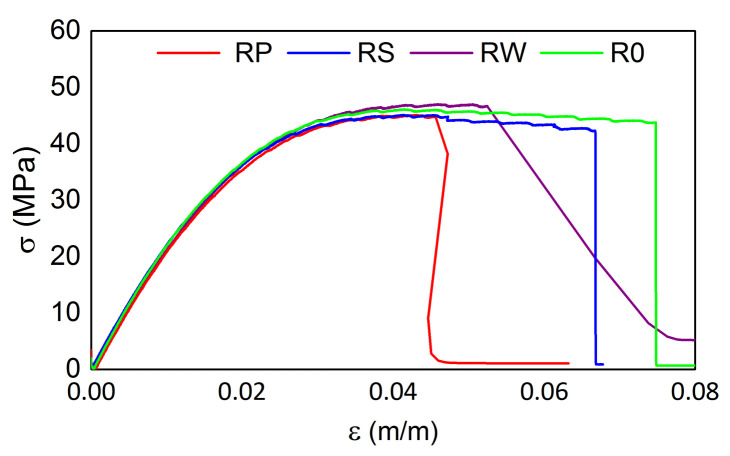
Tensile test results for M3 Crystal samples after exposition on sub-zero temperatures.

The tensile test results from the testing machine for all samples manufactured from M3 Crystal are presented in Figure 18 and Figure 19 for the samples after exposition on elevated and sub-zero temperatures, respectively. The elevated temperatures influence resulted in differences in the tensile strength values among samples and the maximal strength for R0 and RW is ca. 7 MPa higher than for the other two samples. For them, it is equal to ca. 51 MPa. Such a result does not occur for the samples exposed previously to sub-zero temperatures. For such cases, the mean tensile strength value is equal to ca. 46 MPa. Similarly to M3 X, the maximal strain values differ among samples.

Regardless of the material, the embedded sensors’ location, and thermal treatment, the maximal tensile strength values differ slightly. They are also comparable to the tensile strength values for intact samples listed in Table 1 and Table 2, for M3 Crystal and M3 X, respectively. Therefore, it can be concluded that the embedded fibre optic (with FBG sensor) occurrence does not affect the material strength.

**Figure 20:**
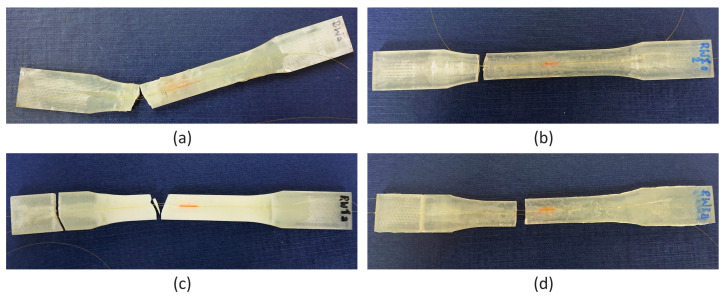
Photographs of samples after the tensile tests: (**a**) M3 X after exposition on elevated temperatures, (**b**) M3 Crystal after exposition on elevated temperatures, (**c**) M3 X after exposition on sub-zero temperatures, and (**d**) M3 Crystal after exposition on sub-zero temperatures.

More detailed analyses are presented for RW samples. The samples were chosen because both of their embedded and attached FBG sensors are located parallel to the main axis of the sample which was the main axis of loading during the tensile test. A set of photographs of RW samples after the tensile tests is presented for comparison in Figure 20. The fracture characteristics depend on the material, while the break location is related to the thermal treatment that probably influenced the material’s internal structure. In both M3 X samples, the embedded fibre optics remained, and still join the parts of the samples. In both cases, a part of the sample in the form of a wedge dropped away during the tensile test. For both M3 Crystal samples, the typical brittle fracture was observed.

**Figure 21:**
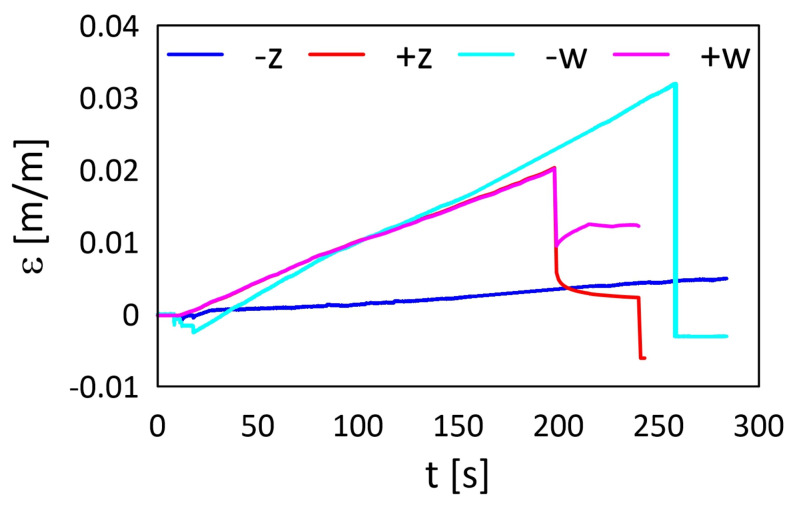
Strain determined for RW samples manufactured from M3 X during the tensile test: *w*—embedded, *z*—attached, ‘+’—after exposure on elevated temperatures, and ‘−’—after exposure on sub-zero temperatures.

**Figure 22:**
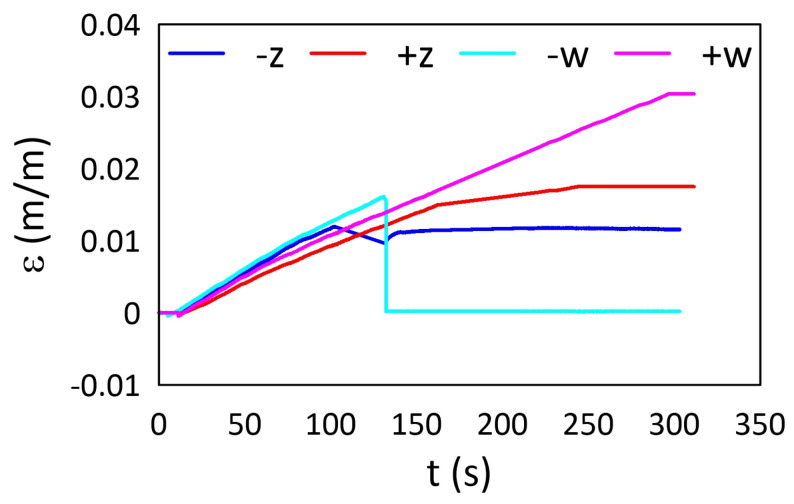
Strain determined for RW samples manufactured from M3 Crystal during the tensile test: *w*—embedded, *z*—attached, ‘+’—after exposing on the elevated temperatures, and ‘−’—after exposing on the sub-zero temperatures.

The strain values from both RWw and RWz sensors determined for the tensile tests are presented in Figure 21 and Figure 22 for M3 X and M3 Crystal, respectively. Both sensors from M3 X sample (Figure 21) exposed previously to elevated temperatures show the same strain trends (values) up to the end of the test. The observed differences after the end of the test are related to the sensors’ condition after the fracture. Contrary to this, RWz sensor from the sample exposed previously to sub-zero temperatures is almost insensitive to the mechanical loading during the test. It was probably due to the attaching process failure, e.g., improper cleaning of the sample surface before the gluing process. The sensor behaviour is similar to extensometer sliding over the sample surface. Such behaviour occurs on a slippery surface of the measured sample and was observed for one of AM polymer samples during the tensile tests. For the majority of sensors (except RWz sensor after the sub-zero treatment), the strain curve shapes show that, after the maximal strain (corresponding to the maximal stress in Figure 16 or Figure 17) the remained strain value in the material is neglected.

Contrary to this, sensors from M3 Crystal samples (Figure 22) indicate changes in the material structures—the strain levels after the end of the test are stable and their values are corresponding to the maximal strain values. The only difference is in RWw sensor after the sub-zero treatment because the sensor was broken during the test.

A comparison of the maximal strain values determined from the testing machine and the FBG sensors are collected in Table 12. For each case of the tensile test, two strain values (from the machine) are given: for RW sample and the mean value for all samples. The achieved results for M3 X material show a good agreement. The percentage difference (between FBG sensors and extensometer) in relation to RW sample is ca. 17% (ca. 20% comparing to the mean value from all samples). The differences can be related to the analysed area of the sample: local (FBG sensors) and global (extensometer).

A finite element method model was used for the determination of strain in the embedded FBG sensor location. The maximal strain value determined numerically was equal to 0.028 m/m, while the tensile strength was equal to 46 MPa. The difference is equal to 10% and it is on an acceptable level. The numerical model details are presented in [37].

The percentage strain differences for M3 Crystal are much higher. The minimum value is for RWw sensor after the sample exposition to elevated temperature and is equal to ca. 36%, while the maximal percentage difference is over 70%. This indicates that the FBG sensors embedded in AM M3 Crystal samples determine the mechanical strain values originated from the tensile force with a very high error. Therefore, it is not recommended to use the sensors for MJP M3 Crystal material for SHM purposes.

## 5. Conclusions

In this paper, a comparison of two AM polymers (M3 X and M3 Crystal) with FBG sensors was presented and discussed. The samples were manufactured using MJP method developed at the IMP PAN. During the manufacturing process, FBG sensors were embedded into the samples and attached to their surfaces using the same UV curable polymer. The aim of the investigation was the determination the influence of the embedded fibre optics with FBG sensors on the material durability.

The experimental investigation was divided into two main parts: thermal and tensile tests. In both cases, the FBG sensors were used for determining the strain values. It was observed that FBG sensors allow the determination of mechanical strain values in both polymers during the thermal tests. For M3 X material, the strain differences between the values determined using the embedded and attached sensors were small—on the $1\times 10^{-5}$ level in relation to the strain values, on the $1\times 10^{-3}$ level for the thermally treated materials, and the measurement accuracy equal to $1\times 10^{-6}$. As expected, the higher differences were for RP samples, where the embedded sensor was located perpendicular to the main axis of the sample. Additionally, the sample width in this area was 12 mm, while the FBG gauge length was equal to 10 mm. For M3 Crystal samples, the strain differences between embedded and attached sensors were higher. Furthermore, the material sensitivity on RH levels was observed, especially by the increase in the mentioned differences with temperature in the elevated temperature test.

For the purpose of determining the relation between materials exposed to elevated or sub-zero temperatures, a comparison of curves (strain vs temperature) for both materials were presented and discussed. Due to M3 Crystal material sensitivity on RH levels and its relatively low heat distortion temperature (56 °C), it was difficult to determine the accurate strain differences between the polymers for the elevated temperatures. Such a problem does not occur for the sub-zero temperatures test. For the test, the mentioned differences strongly depended on RH levels and were equal to ca. $0.64\times 10^{-3}$ and ca. $0.18\times 10^{-3}$ for 20% RH and 95% RH, respectively.

Next, the samples were mechanically tested. The tensile test was performed. The achieved results show that the influence of embedded fibre optics with FBG sensors on the tensile strength of the polymers is neglected. The PW samples manufactured from M3 X have the lower maximal mechanical strain values—this was likely the influence of the embedded sensor location. For M3 X material, the mean tensile strength values were equal to ca. 49 MPa and almost did not depend on the previous thermal treatment. The results were also comparable with the values for the intact material. The tensile strength of M3 Crystal depended on the thermal treatment and the mean tensile strength values for the samples after exposure to sub-zero temperatures was ca. 5 MPa lower than for the other treatment. A comparison of the mechanical strain values determined from FBG sensors and the tensile test machine for RW type samples was presented. It was concluded that FBG sensors embedded in M3 X material show good agreement with the extensometer measurements.

Therefore, the MJP technique can be applied for manufacturing elements with embedded FBG sensors using M3 X material. Such sensors can be also attached to the surfaces of the elements using the same polymer. The sensors can be applied for determining strain values originating from the thermal and mechanical loading.

## Figures and Tables

**Table 1:** Properties of the selected polymeric material—M3 X.

Property	Value
Density at 80 °C (liquid before hardening)	1.04 g/cm^3^
Heat distortion temperature at 0.45 MPa	88 °C
Elongation at break	8.3%
Young’s modulus	2168 MPa
Flexural strength	65 MPa
Tensile strength	53 MPa
Glass transition temperature	65 °C

**Table 2:** Properties of the selected polymeric material—M3 Crystal.

Property	Value
Density at 80 °C (liquid before hardening)	1.02 g/cm^3^
Heat distortion temperature at 0.45 MPa	56 °C
Elongation at break	6.83%
Young’s modulus	1463 MPa
Flexural strength	49 MPa
Tensile strength	47 MPa
Glass transition temperature	65 °C

**Table 3:** Samples dimensions.

Parameter (mm)					
L	D	LO	W	WO	T
70	110	165	12	20	4.20

**Table 4:** Samples symbols.

Symbol	Description/Location of the Embedded FBG Sensor
RW	parallel to the main axis of the sample
RP	perpendicular to the main axis of the sample
RS	on a diagonal of the sample
R0	no sensor

**Table 5:** Sensors symbols.

Sample	FBG Sensor	
	Embedded	Attached
RW	RWw	RWz
RP	RPw	RPz
RS	RSw	RSz
R0		R0z

**Table 6:** Mean strain differences $1\times 10^{-3}$ (m/m) among the samples—all sensors.

Material	T [°C]	All	Without RP	Only RP
M3 X	+	0.147	0.030	0.615
M3 Crystal	+	0.351	0.316	0.488
M3 X	−	0.018	0.015	0.029
M3 Crystal	−	0.059	0.024	0.198

**Table 7:** Mean strain differences $1\times 10^{-3}$ (m/m) among the samples—between embedded (*w*) and attached (*z*) sensors.

Material	T [°C]	20% RH			95% RH		
		All	No RP	RP	All	No RP	RP
M3 X	+	0.058	0.025	0.124	0.384	0.115	0.923
M3 Crystal	+	0.464	0.483	0.426	0.481	0.631	0.180
M3 X	−	0.017	0.025	0.001	0.019	0.016	0.025
M3 Crystal	−	0.120	0.114	0.132	0.043	0.050	0.031

**Table 8:** Strain differences (%) between sample RW and the other samples for embedded (*w*) sensors.

Material	T [°C]	RP		RS	
		20% RH	95% RH	20% RH	95% RH
M3 X	+	1.5	32.7	4.7	2.3
M3 Crystal	+	55.6	61.9	6.1	108.5
M3 X	−	1.5	1.5	0.2	0.8
M3 Crystal	−	10.1	2.1	2.9	0.5

**Table 9:** Strain differences (%) between sample RW and the other samples for attached (*z*) sensors.

Material	T [°C]	R0		RP		RS	
		20% RH	95% RH	20% RH	95% RH	20% RH	95% RH
M3 X	+	1.1	3.5	2.0	8.1	0.3	4.7
M3 Crystal	+	2.5	25.7	50.7	64.1	6.1	3.3
M3 X	−	1.5	1.1	0.3	1.4	0.5	0.9
M3 Crystal	−	0.4	0.3	1.3	0.9	0.1	0.4

**Table 10:** Comparison of the strain curves for both materials after exposition on elevated temperatures.

Difference	Strain $1\times 10^{-3}$ (m/m)			
	FBG Embedded		FBG Attached	
	20% RH	95% RH	20% RH	95% RH
mean	0.576	0.287	0.103	0.225
max or min	0.044	1.283	0.589	0.855
FBG	RPw	RWw	RPz	RPz

**Table 11:** Comparison of the strain curves for both materials after exposition on sub-zero temperatures.

Difference	Strain $1\times 10^{-3}$ (m/m)			
	FBG Embedded		FBG Attached	
	20% RH	95% RH	20% RH	95% RH
mean	0.641	0.183	0.637	0.185

**Table 12:** Comparison of the maximal strain (m/m) in the tensile test.

Material	T [°C]	FBG		Tensile test	
		RWw	RWz	RW	Mean
M3 X	+	0.02016	0.02031	0.02448	0.02557
M3 Crystal	+	0.03034	0.01749	0.04726	0.05635
M3 X	−	0.03195	0.00495	0.04762	0.04442
M3 Crystal	−	0.01611	0.01175	0.05248	0.05991

## Data Availability

Not applicable.
